# Common genetic variants in pre-microRNAs and risk of breast cancer in the North Indian population

**DOI:** 10.3332/ecancer.2014.473

**Published:** 2014-10-20

**Authors:** C Bansal, K L Sharma, Sanjeev Misra, A N Srivastava, Balraj Mittal, U S Singh

**Affiliations:** 1 Pathology Department, King George Medical University, Lucknow 226003, India; 2 Genetics Department, Sanjay Ghandi Postgraduate Institute of Medical Sciences (SGPGIMS), Lucknow 226014, India; 3 Department of Surgical Oncology, King George Medical University, Lucknow 226003, India; 4 Pathology Department, Era’s Medical College and Hospital, Lucknow 226003, India

**Keywords:** breast cancer, MicroRNAs, polymorphisms

## Abstract

**Objective:**

MicroRNAs (miRNAs) are short regulatory RNAs that can modulate gene expression and function as negative regulators. Common genetic variants like single nucleotide polymorphisms (SNPs) in miRNA genes may alter their expression or maturation resulting in varied functional consequences in carcinogenesis. Therefore, we evaluated the genetic variants in pre-miRNAs: hsa-miR-146a G/C (rs2910164), hsa-miR-196a2 C/T (rs11614913), and hsa-miR-499 T>C (rs3746444) for their role in breast cancer susceptibility.

**Study design:**

The study comprised 121 breast cancer patients, 115 with benign breast disease, and 164 controls. The genotypic frequency of miRNA polymorphisms was determined by PCR-RFLP assay. Logistic regression was used for statistical analysis using SPSS Software version 15.0. *In silico* analysis was done using various bioinformatics tools (F-SNP, FAST-SNP).

**Results:**

The heterozygous variant of miR-146a G/C (rs2910164) is associated with the reduced risk of breast cancer at the genotype level as well as at the allele level (*p* < 0.05, OR = 0.5) as compared to controls. On the contrary, no significant difference was observed in the distribution of miR-196a2 C/T (rs11614913) and miR-499 T>C (rs3746444) polymorphisms in any groups both at genotype and allele levels. On the other hand, in multivariate analysis, we found that the miR-196a2 (rs11614913) C>T was associated with an increased risk of breast cancer risk in postmenopausal females (*p* = 0.02, OR = 3.2). We also attempted to find out the risk of malignant breast disease in relation to each of the above SNPs on dividing our data on the basis of benign and malignant status, but no significant difference was observed. *In silico* analysis using F-SNP showed change in transcriptional regulation by miR-146a G/C (rs2910164), miR-196a2 C/T (rs11614913) and miR-499 T>C (rs3746444) variations; the functional score was 0.100, 0.065 and 0.277, respectively.

**Conclusion:**

The results of the present study demonstrate that miR-146a G/C (rs2910164) polymorphism is associated with reduced genetic susceptibility to breast cancer. However, multivariate analysis showed as miR-196a2 (rs11614913) C>T to be associated with increased risk of breast cancer risk in postmenopausal females. Further multicentric studies involving a large number of cases need to be carried out to strengthen the present results.

## Introduction

Breast cancer is the most frequently diagnosed cancer among women. It is the leading cause of cancer death in the less-developed countries [1]. According to the National Cancer Registry Programme, cancers of the uterine cervix and breast are the two leading cancer sites among Indian women. Published reports from different cancer registries in India indicate rising trends in breast cancer incidence. Age-adjusted rate incidence has shown a rise from 19.4 (year 1982) to 27.72 (year 2008) [2, 3].

Many environmental and genetic factors are known to play an important role in the breast cancer genesis and prognosis. The BRCA1 and BRCA2 mutations together account for about 20–25% of hereditary breast cancers [4] and about 5–10% of all breast cancers [5]. However, more studies are needed to identify other genes having an impact on breast cancer risk and prognosis that may play a major role in risk prediction. Breast cancer originates from breast tissue, usually from the inner lining of the milk ducts or the lobules that supply the ducts with milk. Cancers originating from ducts are known as ductal carcinomas, while those originating from lobules are known as lobular carcinomas [6].

MicroRNAs (miRNAs) are small, non-coding, approximately 22-nucleotide miRNAs that regulate gene expression [7]. They function either as tumour suppressors or oncogenes through sequence-specific base-pairing with target mRNAs [8, 9]. Common single-nucleotide polymorphisms (SNPs) in miRNAs may change their properties through altering miRNA expression and/or maturation, and thus they may have an effect on thousands of target messenger RNAs (mRNAs), resulting in diverse functional consequences. However, it remains largely unknown whether miRNA SNPs may alter cancer susceptibility. Over the past 2–3 years, several reports have shown that singlenucleotide polymorphisms (SNPs) in the precursor of miRNAs (pre-miRNAs) particularly miR-146a G/C, miR-196a2 C/T and miR-499 T>C polymorphisms affect maturation/expression of respective mature miRNA and are studied in several cancers including carcinoma of the breast, urinary bladder, cervical, head and neck, gastric, and lung [9–12].

So, this present case control association study was designed to evaluate the role of pre-miRNA genetic variations miR-196a2 C/T (rs11614913), miR-146a G/C (rs2910164) and miR-499 T>C (rs3746444) polymorphisms in susceptibility to breast cancer.

## Materials and methods

### Study subjects

In the present case control study, we recruited 121 breast cancer (CaB) patients, 115 with benign breast disease, and 164 healthy controls. The cases and the controls included in the present study were unrelated and were of similar ethnicity, i.e., North Indian. The subjects were recruited from the department of surgical oncology, King George Medical University, Lucknow, India. The healthy controls were recruited from unrelated individuals within the general population. The inclusion criteria for the controls were: absence of prior history of cancer or precancerous lesions and absence of any chronic disease/malignancy and were frequency-matched to cancer cases for age, gender, and ethnicity. Written informed consent was obtained from all the individuals. The study protocols and the work were approved by the institutional ethical committee, and the authors followed the norms of the World’s Association Declaration of Helsinki.

### Genotyping

Blood samples (3.0 ml) from cases of breast cancer, benign breast disease, and control subjects were collected in ethylene diamine tetra-acetic acid (EDTA) vials. Genomic DNA extraction from peripheral blood leucocytes was carried out using salting out method as described by Miller *et al* [13]. The genotyping of miR-146a G/C, miR-196a2 C/T, and miR-499 T>C polymorphisms were performed through PCR-RFLP, as described previously [14].

### Statistical analysis

Statistical analysis was done using SPSS version 16.0 (SPSS, Chicago, Illinois USA). Descriptive statistics of patients and controls were presented as the mean and standard deviations for continuous measures, while frequencies and percentages were used for categorical measures. The null hypothesis that the Hardy–Weinberg equilibrium holds was tested using a chi-squared test for deviation from Hardy–Weinberg equilibrium. Binary logistic regression was performed to find out the risk genotype. Association was expressed as odds ratios (OR) with 95% confidence intervals (CI). The association was considered to be significant when the P-value was <0.05. Bioinformatics analysis was done by using bioinformatics tools FAST-SNP (http://fastsnp.ibms.sinica.edu.tw) and F-SNP http://compbio.cs.queensu.ca/F-SNP/ [15–16].

## Results

### Characteristic Profile of the Study Subjects

A total of 400 study subjects was recruited in this study, including 115 with benign breast disease, 121 breast cancer patients and 164 controls. All cases were biopsy-/cytology-proven for benign or malignant disease. The mean ages of benign and malignant cases were 36 and 58 years, respectively. Among malignant cases, 7.5% of the cases had metastasis. Clinicopathological profile data of malignant cases are shown in Table 1.

#### Clinico-pathological profile of breast carcinoma patients

The most common histological type was infiltrating duct carcinoma (IDC - 97.7%), with the presence of *in situ* Carcinoma in 38.5%. The majority had MRB Grade II tumour (66.2%). Lymph nodes were positive in 64.6%. Tumour infiltrating lymphocytes were seen in 35.4% of cases. The most common stage was T2N1M0. Hormone receptor negative (triple negative) cases were 24.6% (Table 2).

#### Distribution of studied polymorphisms in controls

The distribution of miR-146a G/C (rs2910164), miR-196a2 C/T (rs11614913) and miR-499 T>C (rs3746444) polymorphisms is shown in Table 3. The observed genotype frequencies of all the studied polymorphisms in controls were in accordance with the Hardy-Weinberg equilibrium (*p* > 0.05).

#### Association of miR-146a G/C (rs2910164), miR-196a2 C/T (rs11614913) and miR-499 T>C (rs3746444) polymorphisms with breast carcinoma versus controls

Table 3 shows the risk of breast cancer in relation to each of the SNPs of miR-146a G/C (rs2910164), miR-196a2 C/T (rs11614913) and miR-499 T>C (rs3746444) when compared with controls. On comparing the genotype frequency miR-146a G/C, distribution in CaB patients with that of controls, the heterozygous variant [GC] and [C] allele showed statistically negative significant risk with CaB (*p* = 0.013; [OR] = 0.5; *p* = 0.01; [OR] = 0.6). On the contrary, no significant difference was observed in the distribution of miR-196a2 C/T (rs11614913) and miR-499 T>C (rs3746444) polymorphisms in any groups both at the genotype and allele levels.

#### Association of miR-146a G/C (rs2910164), miR-196a2 C/T (rs11614913) and miR-499 T>C (rs3746444) polymorphisms in breast disease on stratifying data on the basis of benign and malignant status

Table 4 shows the risk of malignant breast disease in relation to each of the SNPs of miR-146a G/C (rs2910164), miR-196a2 C/T (rs11614913) and miR-499 T>C (rs3746444) after stratifying our data on the basis of benign and malignant status. Statistically significant association was not found.

#### Co-relation of pre-microRNA polymorphisms miR-146a G/C (rs2910164), miR-196a2 C/T (rs11614913) and miR-499 T>C (rs3746444) with clinical characteristics (tumour stage and lymph node involvement) in breast cancer patients

The study did not find any significant association of all the selected variants miR-146a G/C (rs2910164), miR-196a2 C/T (rs11614913) and miR-499 T>C (rs3746444) on the basis of segregation of CaB patients according to tumour stage and lymph node involvement (data not shown).

#### Co-relation of miR-146a G/C (rs2910164), miR-196a2 C/T (rs11614913) and miR-499 T>C (rs3746444) polymorphisms with hormonal receptor status (ER/PR/HER2) and menopausal status

The present study did not find any significant association of all selected variants miR-146a G/C (rs2910164), miR-196a2 C/T (rs11614913) and miR-499 T>C (rs3746444) on the basis of ER, PR, and HER2 presence or absence in case only analyses. In multivariate analysis, miR-196a2 (rs11614913) C>T was associated with increased risk of breast cancer in postmenopausal group (*p* = 0.02, [OR] = 3.2] (Data not shown), whereas no association of other variants (*p* > 0.05) was found. We did not find any association in any of the above parameters on comparing the benign and malignant groups.

### Bioinformatic analysis

*In silico* analysis using F-SNP showed change in transcriptional regulation by miR-146a G/C (rs2910164), miR-196a2 C/T (rs11614913) and miR-499 T>C (rs3746444) variations (Functional score 0.101, 0.065 and 0.277, respectively (Table 5).

## Discussion

The miRNAs are non-coding RNA known to regulate the gene expression of several target genes, which may later be involved in the carcinogenesis process [17–18]. They are well known for their imperative function in diverse cellular processes, such as proliferation, differentiation, apoptosis, and various diseases including cancer [19–23]. Around 30% of the genes in human beings are regulated by miRNAs, and they act as endogenous repressors of target genes, including tumour suppressor genes, such as BRCA1-2, p53 and PTEN [24]. Recent studies have shown that genetic variants in miRNA affect progression, diagnosis and prognosis of various malignancies [14, 25–28].

The present case-control study evaluated the potential association of three SNPs (rs2910164, rs11614913 and rs3746444) in pre-miRNAs with 115 benign breast disease and 121 breast cancer patients, and 164 controls in a North Indian population. It was found that the heterozygous variant of miR-146a G/C is associated with a statistically significant reduced risk of breast cancer. On the contrary, no significant difference was observed in the distribution of miR-196a2 C/T (rs11614913) and miR-499 T>C (rs3746444) polymorphisms in any groups both at genotype and allele levels.

Mir-146a regulates many genes like IRAK1, ST7L, BCORL1, CARD10, and MMP16 [14]. Target genes of mir-499 are transcription factors include SRY-box 6 (SOX-6), SOX5, DDX1, LIN28B, PDCD4, E2F3 [14]. Some of these target genes like HOXC8, HOXA7, HOXB7, LRP1B, p27 (regulated by mir-196a2), IRAK1, CARD10 (targets genes of mir-146), SOX6, PDCD4 (target genes of mir-499), and PABC1 (regulated by mir-423) (http://www.microrna.org/) are deregulated in many cancers [14]. Hence, various single nucleotide variations (SNPs) in pre-miRNA may affect the maturation/expression of miRNA, consequently may alter the miRNA targeted oncogenes/tumour suppressor genes expression, and thus may affect the susceptibility/prognosis of cancer.

Several previous studies have been reported in the literature showing the influence of miRNA 146a in various cancers. However, there are very few studies in the literature showing the role of miRNA 146a C>G polymorphism in breast cancer. A recent study reported that the rs2910164 C>G in pre-miR-146a may contribute to genetic susceptibility to lung cancer [29]. However, another meta-analytic study revealed that miR-146a (rs2910164) polymorphism has no major role in genetic susceptibility to hepatocellular carcinogenesis [30]. A study by Mao *et al* showed the association between miR-146a genetic variants and colorectal cancer [31]. Thus, microRNA-146a is associated with various cancers and its role in genetic susceptibility varies.

A polymorphism study by Hou *et al* has shown that miR-499 influences the expression levels of miR-499a-5p during the tumourogenesis of oral squamous cell carcinoma. The authors have concluded that miR-499a may contribute to an increased risk of BQ-related oral sub mucous fibrosis, but a decreased risk of oral squamous cell carcinoma [32]. A meta-analytic study by Xu *et al* has clarified that three polymorphisms in microRNAs have different effects on cancer risk in the Asian population [33]. Evidence from the published literature clearly shows that TT genotype of rs11614913 polymorphism is associated with decreased cancer risk, especially for colorectal carcinoma and lung cancer among the Korean and North Indian population. Moreover, rs2910164 C allele was associated with decreased overall cancer risk especially for hepatocellular carcinoma, cervical cancer, and prostate cancer risk among the Chinese population, whereas rs3746444 G allele was a risk factor among the Chinese population, especially for breast cancer [33].

We also tried to find out the risk of malignant breast disease in relation to each of the above SNPs on dividing our data on the basis of benign and malignant status but no significant difference was observed. The correlation of pre-micro RNA polymorphisms mir-146a, mir-196a2 and mir-499 with clinicopathological parameters (lymph node and tumour stage) did not find any significant difference. In the multivariate analysis, the miR-196a2 C/T (rs11614913) was associated with increased risk of breast cancer risk in postmenopausal group, but no association was found when comparing the benign and malignant group.

An *In silico* analysis using FSNP showed a change in transcriptional regulation by miR-146a G/C (rs2910164), miR-196a2 C/T (rs11614913) and miR-499 T>C (rs3746444) variations (Functional score 0.101, 0.065 and 0.277, respectively).

However, the limitations and strengths of our study need to be mentioned. The disease was histopathologically confirmed in all the cases, and the controls were matched for age, gender, and ethnicity. However, the sample size in the present study is sufficient to yield 80% power, but is limited in subgroup analysis.

## Conclusion

Our study shows that miR-146a G/C (rs2910164) may confer a reduced risk of breast cancer. We have found in a multivariate analysis that miR-196a2 (rs11614913) C>T is associated with increased risk of breast cancer in the postmenopausal group (*p* = 0.02). However, the mechanisms underlying this association need to be further explored. Further studies investigating the role of pre-miRNA genetic variants in breast cancer and replication of the present study in diverse ethnic groups are needed to better understand the pathobiology of breast cancer.

## Conflict of interest

The authors have no conflicts of interest to declare.

## Figures and Tables

**Table 1.** Clinicopathological profile of breast carcinoma patients.

Variables	Status	No. (%)
Age group	<40	40 (33.0)
	>40	81 (67.0)
Side	Left	57 (46.9)
	Right	64 (53.1)
Menopausal status	Pre menopausal	82 (67.7)
	Post menopausal	39 (32.3)
Tumour size	< or = 2	20 (16.2)
	2–5	72 (60)
	>5	29 (23.8)
Tumour type	IDC	118 (97.5)
	ILC	3 (2.5)
In situ component	Absent	75 (61.6)
	Present	46 (38.4)
MRB grade	I	11 (9.2)
	II	80 (66.2)
	III	30 (24.6)
Lymph node	Absent	43 (35.4)
	Present	78 (64.6)
Skin infiltration	Absent	107 (89.2)
	Present	14 (10.8)
Metastasis	Absent	112 (92.5)
	Present	9 (7.5)
Intratumoural and peritumoural lymphocytes	Absent	78 (64.6)
	Present	43 (35.4)

**Table 2.** Hormone receptor status of breast carcinoma patients.

Hormonal Receptor	Status	No. (%)
ER	Negative	60 (49.2)
	Positive	61 (50.8)
PR	Negative	70 (57.7)
	Positive	51 (42.3)
HER2	Negative	72 (59.23)
	Positive	49 (37.69)
ER/ PR/ HER2	Triple negative	29 (24.6%)

**Table 3.** Association of miR-146a G/C (rs2910164), miR-196a2 C/T (rs11614913) and miR-499 T>C (rs3746444) polymorphisms with CaB verses controls.

Genotype/Allele	Controls *n* = 164 (%)	CaB *n* = 121(%)	OR (95%CI)	*p*-value
**miR-146a G/C (rs2910164)**				
*GG*	84 (51.2%)	82 (67.8%)	1 (Reference)	-
*GC*	72 (43.9%)	35 (28.9%)	**0.5 (0.31–0.87**	0.013^*^
*CC*	8 (4.9%)	4 (3.4%)	0.53 (0.15–1.85)	0.32
*G*	240 (73.17%)	199 (82.23%)	1 (Reference)	-
*C*	88 (26.83%)	43 (17.77%)	**0.6 (0.4–0.8)**	0.01^*^
**miR-196a2 C/T (rs11614913)**				
*CC*	85 (51%)	68 (55.9%)	1 (Reference)	-
*CT*	59 (36%)	41 (33.9%)	1.0 (0.6–1.7)	0.8
*TT*	21 (13%)	12 (10.2%)	0.9 (0.4–2.0)	1.0
*C*	229 (69.39%)	177 (73.14%)	1 (Reference)	-
*T*	101 (30.61%)	65 (26.86%)	1.2 (0.8–1.7)	0.3
**miR-499 T>C (rs3746444)**				
*TT*	106 (64.6%)	80 (66.4%)	1 (Reference)	-
*TC*	43 (26.2%)	30 (24.4%)	1.0 (0.6-1.8)	0.89
*CC*	15 (9.14%)	11 (9.2%)	1.0 (0.4-2.2)	1.0
*T*	255 (77.74%)	190 (78.51%)	1 (Reference)	-
*C*	73 (22.26%)	52 (21.49%)	1.0 (0.7-1.5)	0.8

CaB-breast cancer,

OR-Odds Ratio,

CI-Confidence Interval.

Significant Values are given in bold

* refer to significant *p*-value

**Table 4.** Association of miR-146a G/C (rs2910164), miR-196a2 C/T (rs11614913) and miR-499 T>C (rs3746444) polymorphisms with benign breast disease.

Genotype/Allele	Benign *n* = 115 (%)	CAB *n* = 121 (%)	OR (95%CI)	*p-value*
**miR-146a G/C (rs2910164)**				
*GG*	66 (57%)	82 (67.8%)	1 (Reference)	-
*GC*	45 (39.5%)	35 (28.9%)	0.6 (0.3–1.06)	0.08
*CC*	4 (3.5%)	4 (3.4%)	0.79 (0.19–3.2)	0.74
*G*	177 (76.96%)	199 (82.23%)	1 (Reference)	-
*C*	53 (23.04%)	43 (17.77%)	1.3 (0.8–2.1)	0.17
**miR-196a2 C/T (rs11614913)**				
*CC*	57 (49.57%)	68 (55.9%)	1 (Reference)	-
*CT*	44 (38.26%)	41 (33.9%)	0.76 (0.4–1.3)	0.33
*TT*	14 (12.17%)	12 (10.2%)	0.68 (0.2–1.6)	0.38
*C*	158 (68.7%)	177 (73.14%)	1 (Reference)	-
*T*	72 (31.3%)	65 (26.86%)	1.2 (0.8–1.8)	0.31
**miR-499 T>C (rs3746444)**				
*TT*	75 (65.12%)	80 (66.4%)	1 (Reference)	-
*TC*	29 (25.22%)	30 (24.4%)	1.0 (0.5–1.8)	1.0
*CC*	11 (9.57%)	11 (9.2%)	1.0 (0.4–2.5)	1.0
*T*	179 (77.83%)	190 (78.51%)	1 (Reference)	-
*C*	51 (22.17%)	52 (21.49%)	1.0 (0.6–1.6)	0.9

CaB-breast cancer,

OR-Odds Ratio,

CI-Confidence Interval

**Table 5.** *In silico* analysis of selected variants.

Result of F SNP				
Genetic Variation	Functional Category	Prediction Tool	Prediction Result	FS score
miR-146aG/C (rs2910164)	Transcriptional regulation	GoldenPath	Exist	0.101
miR-196a2C/T (rs11614913)	Transcriptional Regulation	GoldenPath	Exist	0.065
miR-499T>C (rs3746444)	Transcriptional regulation	GoldenPath TFSearch	Exist Not Changed	0.277
